# Supplementary material for: Resolving the full spectrum of human genome variation using Linked-Reads
Source: Genome Res. 2019 Apr;29(4):635–45. doi: 10.1101/gr.234443.118 (PMC6442396; doi:10.1101/gr.234443.118)
Supplement: Supplemental Material [file supp_gr.234443.118_Supplemental_longranger-2.2.zip › longranger-2.2.tar.bz2!/lr2.2-public/lib/stan/cmdstan/stan/doc/api/html/agrad_2rev_2matrix_2dot__self_8hpp_source.html]

Stan: src/stan/agrad/rev/matrix/dot\_self.hpp Source File


|  |  |
| --- | --- |
|  | Stan  2.5.0  probability, sampling & optimization |


- Main Page
- Related Pages
- Namespaces
- Classes
- Files

- File List
- File Members

All Classes Namespaces Files Functions Variables Typedefs Enumerations Enumerator Friends Macros Pages

- src
- stan
- agrad
- rev
- matrix

dot\_self.hpp

Go to the documentation of this file.

1 #ifndef STAN\_\_AGRAD\_\_REV\_\_MATRIX\_\_DOT\_SELF\_HPP

2 #define STAN\_\_AGRAD\_\_REV\_\_MATRIX\_\_DOT\_SELF\_HPP

3

4 #include <vector>

5 #include <stan/math/matrix/Eigen.hpp>

6 #include <stan/math/matrix/typedefs.hpp>

7 #include <stan/math/error\_handling/matrix/check\_vector.hpp>

8 #include <stan/agrad/rev/var.hpp>

9 #include <stan/agrad/rev/matrix/typedefs.hpp>

10

11 namespace stan {

12  namespace agrad {

13

14  namespace {

15  class dot\_self\_vari : public vari {

16  protected:

17  vari\*\* v\_;

18  size\_t size\_;

19  public:

20  dot\_self\_vari(vari\*\* v, size\_t size)

21  : vari(var\_dot\_self(v,size)),

22  v\_(v),

23  size\_(size) {

24  }

25  template<typename Derived>

26  dot\_self\_vari(const Eigen::DenseBase<Derived> &v) :

27  vari(var\_dot\_self(v)), size\_(v.size()) {

28  v\_ = (vari\*\*)memalloc\_.alloc(size\_\*sizeof(vari\*));

29  for (size\_t i = 0; i < size\_; i++)

30  v\_[i] = v[i].vi\_;

31  }

32  template <int R, int C>

33  dot\_self\_vari(const Eigen::Matrix<var,R,C>& v) :

34  vari(var\_dot\_self(v)), size\_(v.size()) {

35  v\_ = (vari\*\*) memalloc\_.alloc(size\_ \* sizeof(vari\*));

36  for (size\_t i = 0; i < size\_; ++i)

37  v\_[i] = v(i).vi\_;

38  }

39  inline static double square(double x) { return x \* x; }

40  inline static double var\_dot\_self(vari\*\* v, size\_t size) {

41  double sum = 0.0;

42  for (size\_t i = 0; i < size; ++i)

43  sum += square(v[i]->val\_);

44  return sum;

45  }

46  template<typename Derived>

47  double var\_dot\_self(const Eigen::DenseBase<Derived> &v) {

48  double sum = 0.0;

49  for (int i = 0; i < v.size(); ++i)

50  sum += square(v(i).vi\_->val\_);

51  return sum;

52  }

53  template <int R, int C>

54  inline static double var\_dot\_self(const Eigen::Matrix<var,R,C> &v) {

55  double sum = 0.0;

56  for (int i = 0; i < v.size(); ++i)

57  sum += square(v(i).vi\_->val\_);

58  return sum;

59  }

60  virtual void chain() {

61  for (size\_t i = 0; i < size\_; ++i)

62  v\_[i]->adj\_ += adj\_ \* 2.0 \* v\_[i]->val\_;

63  }

64  };

65  }

76  template<int R, int C>

77  inline var dot\_self(const Eigen::Matrix<var, R, C>& v) {

78  stan::math::check\_vector("dot\_self(%1%)",v,"v",(double\*)0);

79  return var(new dot\_self\_vari(v));

80  }

81

87  template<int R,int C>

88  inline Eigen::Matrix<var,1,C>

89  columns\_dot\_self(const Eigen::Matrix<var,R,C>& x) {

90  Eigen::Matrix<var,1,C> ret(1,x.cols());

91  for (size\_type i = 0; i < x.cols(); i++) {

92  ret(i) = var(new dot\_self\_vari(x.col(i)));

93  }

94  return ret;

95  }

96

97

98  }

99 }

100 #endif

stan::agrad::columns\_dot\_self

Eigen::Matrix< fvar< T >, 1, C > columns\_dot\_self(const Eigen::Matrix< fvar< T >, R, C > &x)

**Definition:** columns\_dot\_self.hpp:15

stan::agrad::dot\_self

fvar< T > dot\_self(const Eigen::Matrix< fvar< T >, R, C > &v)

**Definition:** dot\_self.hpp:16

typedefs.hpp

typedefs.hpp

v\_

vari \*\* v\_

**Definition:** dot\_self.hpp:17

stan::agrad::square

fvar< T > square(const fvar< T > &x)

**Definition:** square.hpp:15

stan::agrad::memalloc\_

memory::stack\_alloc memalloc\_

**Definition:** var\_stack.cpp:16

stan::math::check\_vector

bool check\_vector(const char \*function, const Eigen::Matrix< T, R, C > &x, const char \*name, T\_result \*result)

**Definition:** check\_vector.hpp:14

stan::agrad::sum

fvar< T > sum(const Eigen::Matrix< fvar< T >, R, C > &m)

**Definition:** sum.hpp:14

stan::agrad::size\_type

Eigen::Matrix< double, Eigen::Dynamic, Eigen::Dynamic >::Index size\_type

**Definition:** typedefs.hpp:14

var.hpp

Eigen.hpp

stan::memory::stack\_alloc::alloc

void \* alloc(size\_t len)

Return a newly allocated block of memory of the appropriate size managed by the stack allocator...

**Definition:** stack\_alloc.hpp:168

check\_vector.hpp

stan::agrad::var

Independent (input) and dependent (output) variables for gradients.

**Definition:** var.hpp:27

stan::math::size

int size(const std::vector< T > &x)

**Definition:** size.hpp:11

size\_

size\_t size\_

**Definition:** dot\_self.hpp:18


---

[ Stan Home Page ]

*© 2011–2014,
Stan Development Team.*
